# Microbial Biosurfactant as an Alternate to Chemical Surfactants for Application in Cosmetics Industries in Personal and Skin Care Products: A Critical Review

**DOI:** 10.1155/2023/2375223

**Published:** 2023-04-13

**Authors:** Arun Karnwal, Seweta Shrivastava, Abdel Rahman Mohammad Said Al-Tawaha, Gaurav Kumar, Rattandeep Singh, Anupam Kumar, Anand Mohan, Yogita, Tabarak Malik

**Affiliations:** ^1^Department of Microbiology, School of Bioengineering & Biosciences, Lovely Professional University, Phagwara, Punjab, India; ^2^Department of Plant Pathology, School of Agriculture, Lovely Professional University, Phagwara, Punjab, India; ^3^Department of Biological sciences Al Hussein Bin Talal University Ma'an, P.O. Box 20, Jordan; ^4^Department of Molecular Biology and Genetic Engineering, School of Bioengineering & Biosciences, Lovely Professional University, Phagwara, Punjab, India; ^5^Department of Biotechnology, School of Bioengineering & Biosciences, Lovely Professional University, Phagwara, Punjab, India; ^6^Department of Biomedical Sciences, Institute of Health, Jimma University, Ethiopia

## Abstract

Cosmetics and personal care items are used worldwide and administered straight to the skin. The hazardous nature of the chemical surfactant utilized in the production of cosmetics has caused alarm on a global scale. Therefore, bacterial biosurfactants (BS) are becoming increasingly popular in industrial product production as a biocompatible, low-toxic alternative surfactant. Chemical surfactants can induce allergic responses and skin irritations; thus, they should be replaced with less harmful substances for skin health. The cosmetic industry seeks novel biological alternatives to replace chemical compounds and improve product qualities. Most of these chemicals have a biological origin and can be obtained from plant, bacterial, fungal, and algal sources. Various biological molecules have intriguing capabilities, such as biosurfactants, vitamins, antioxidants, pigments, enzymes, and peptides. These are safe, biodegradable, and environmentally friendly than chemical options. Plant-based biosurfactants, such as saponins, offer numerous advantages over synthetic surfactants, i.e., biodegradable, nontoxic, and environmentally friendly nature. Saponins are a promising source of natural biosurfactants for various industrial and academic applications. However, microbial glycolipids and lipopeptides have been used in biotechnology and cosmetics due to their multifunctional character, including detergency, emulsifying, foaming, and skin moisturizing capabilities. In addition, some of them have the potential to be used as antibacterial agents. In this review, we like to enlighten the application of microbial biosurfactants for replacing chemical surfactants in existing cosmetic and personal skincare pharmaceutical formulations due to their antibacterial, skin surface moisturizing, and low toxicity characteristics.

## 1. Introduction

The human skin is the largest organ in the body and has a complex structure. The primary function of the skin is to act as a protective barrier that prevents excessive loss of bodily fluids and blocks the entry of harmful substances and pathogens from the external environment [1, 2]. The histological structure of human skin may be divided into three distinct layers: innermost hypodermis, middle layer dermis, and outermost epidermis [3]. The skin's functionality is greatly impacted by its different layers. The epidermal cells and skin bacteria have a complex relationship that enables various types of mutually beneficial microbes to inhabit the skin. These microorganisms can thrive in the skin's moist, dry, and oily regions located on the surface through selective colonization [4, 5]. Numerous microorganisms that live on or in the skin confer significant advantages on their host. Some of these bacteria contribute to the activation of the innate immune system, while others produce antimicrobial compounds (such as bacteriocins) that prevent infections and pathogens' proliferation. Maintaining a healthy skin microbiome is crucial for overall human health, as the microflora need to be able to withstand environmental stressors such as naturally occurring toxins and the use of specific skincare and grooming products. Therefore, it is important to ensure that the microflora is provided within the appropriate environmental parameters [4, 6].

Developing formulations that improve the skin's barrier function, inhibit the growth of harmful agents, moisturize and cleanse body surfaces, and safeguard the skin, its microflora, and associated cells is a routine practice in the cosmetics and personalized care product industry. All these benefits contribute to improving the skin's overall health. Researchers and manufacturers of personalized skincare products have conducted substantial studies to identify unique and potentially beneficial chemicals that may be put into their formulae to achieve the above-mentioned goals [1, 2, 7]. Presently, chemical surfactants are used as emulsifiers and foaming agents by a wide variety of personal skincare product producers [1, 8]. It was supposed that 50% of the chemical surfactants used for commercial applications presently come from the petrochemical industry, and as a result, they are produced from sources that are not sustainable [9]. It has been reported that these chemical surfactants in various formulations could be harmful to the microbiota of the skin and the skin itself [10]. Some of these chemicals affect the skin flora, which can lead to a negative effect on the skin, i.e., itching, irritation, and allergy, due to interaction with lipids and proteins available in the cell membrane of the epidermis. Furthermore, the excessive usage and high concentration of chemical surfactants have been reported to cause solubilization of the epidermis and intracellular lipids [11, 12]. This impaired the skin's structural integrity and barrier functionality. Because of these factors, there has been a need to replace chemical surfactants with other biological-origin sustainable chemicals without causing any negative impact on the skin. Therefore, the manufacture of these compounds should rely on renewable and sustainable resources while also ensuring they are nontoxic, biodegradable, and safe for human skin. This approach would minimize adverse effects on the environment and consumer health [13, 14].

**Figure 1:**
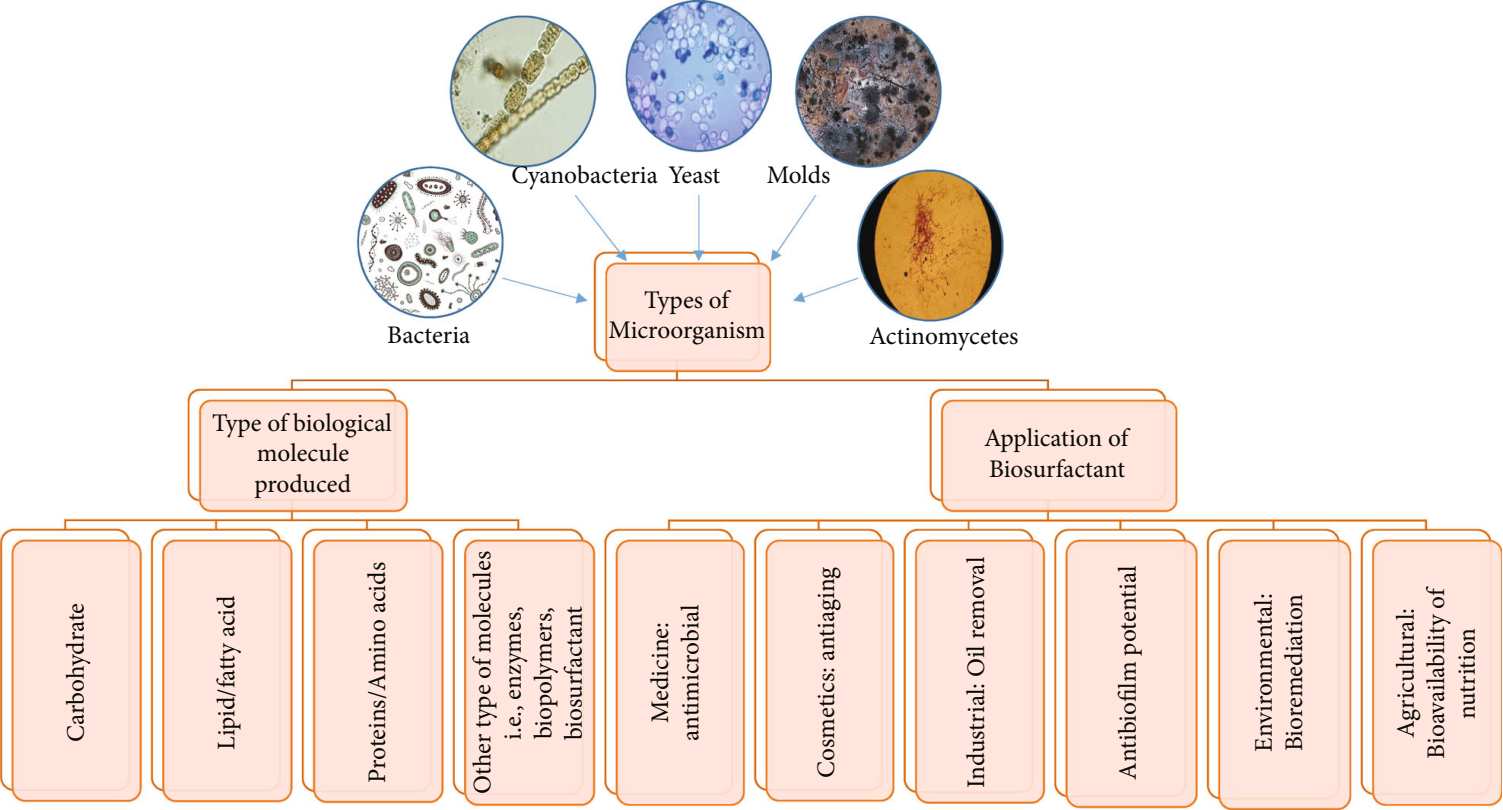
Types of microorganisms and produced biomolecules used as biosurfactant and biosurfactant application in different areas.

Biosurfactants are biological origin surface-active chemicals synthesized by plants, bacteria, yeast, or filamentous fungi as secondary metabolites [15] as presented in figure 1. They are distinguished from traditional surfactants by their biological origin and do not have any added chemical synthesis step during its production. According to Subsanguan et al. [16], biosurfactants typically exist in a neutral or anionic state in their natural form. Conversely, compounds that contain amine groups are classified as cationic (Figure 1). The different structures of biosurfactants can be attributed to the microbiological source from which they are derived, the substrates used for their cultivation, and the specific growth conditions employed [17]. Biosurfactants like rhamnolipids, sophorolipids, mannosyl-erythritol lipids (MELs), and surfactin are some examples of biosurfactants that are the subject of substantial research.

**Figure 2:**
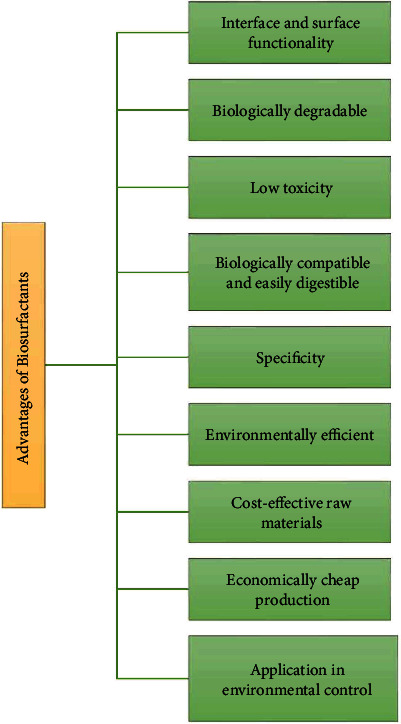
Advantages of biosurfactants compared to chemical surfactants.

Biosurfactants have several benefits over their synthetic counterparts [13, 18]. These advantages include the following (Figure 2):

(1) Less or nontoxicity
(2) Detergency function
(3) Moisturizing
(4) Biodegradable
(5) Emulsification
(6) Safe for human skin applications
(7) Lowering surface tension
(8) Stable in various physical environments (salinity, temperature, and pH), and
(9) Biosynthesis using low-cost and renewable raw materials
  - *Interface and surface functionality*. An efficient surfactant must lower the surface tension of water and interfacial tension with hexadecane to 35 from 75 dyne/cm and 40 to 1, respectively. Surfactin biosurfactants lower the water surface tension and interfacial tension of water/hexadecane below 25 dyne/cm and 1 dyne/cm, respectively, making it a potent biosurfactant [19].
  - *Biologically degradable*. Biosurfactants are suitable for biodegradation/biosorption because microorganisms break them down faster than their synthetic counterparts. Surfactants made from synthetic chemicals pose a risk to the environment; as a result, biosurfactants that can be broken down naturally are seen as a more environmentally friendly alternative [20, 21].
  - *Low toxicity*. Biosurfactants are less hazardous than chemical surfactants. Additionally, they are 50% more efficient at reducing the test population than their synthetic equivalent.
  - *Biologically compatible and easily digestible*. This makes them useful in cosmetics, medicines, and therapeutic dietary supplements [20].
  - *Specificity*. Biosurfactants are multifaceted macromolecules containing various functional groups, each of which is responsible for the response specificity of the biosurfactant. This is important for certain detoxifying contaminants, demulsifying commercial emulsions, and skin care, medicinal, and food applications [22].
  - *Environmentally efficient*. Various biosurfactants function effectively under varied environments, i.e., elevated temperature, broad pH range, and ionic concentration. *Bacillus licheniformis* produces lichenysin, which can function at 50°C, 4.5–9.0 pH, 50 gm/l NaCl concentration, and 25 gl/l Ca^+2^ concentration [23].
  - *Cost-effective raw materials*. Cheap natural resources can be used to synthesize biosurfactants. Biosurfactants require high carbon content-based substrate, i.e., hydrocarbons, carbohydrates, and lipids [24].
  - *Economically cheap production*. Based on the individual usage application, a substantial amount of biosurfactants can be produced from waste and industrial residues, making them cheap compared to chemical surfactants [25, 26].
  - *Application in environmental control*. Biosurfactants have several applications in industry, including emulsion stabilization, oil spill cleanup, wastewater treatment, and bioremediation [18].

Biosurfactants have attracted significant attention from various sectors, including the environmental, oil, agriculture, textile, food, cosmetics, medicine, and pharma industries, in recent decades due to their versatile properties [27–29]. However, microbial biosurfactants' potential applications in medical, cosmetic, and personalized care products have received little attention. Despite this, extensive research has been conducted on biosurfactant production, characterization, and usage in different sectors, i.e., environmental sustainability, oil industries, food, and agribusiness [28, 30]. The aim of this review is to emphasize the advantages of substituting chemical surfactants with biosurfactants in personalized cosmetic and skincare products. The focus is particularly on the antibacterial properties of biosurfactants, their ability to moisturize the skin, and their reduced toxicity.

## 2. Potential Effects of Chemical Surfactants in Personal Care Products on Skin Health

**Figure 3:**
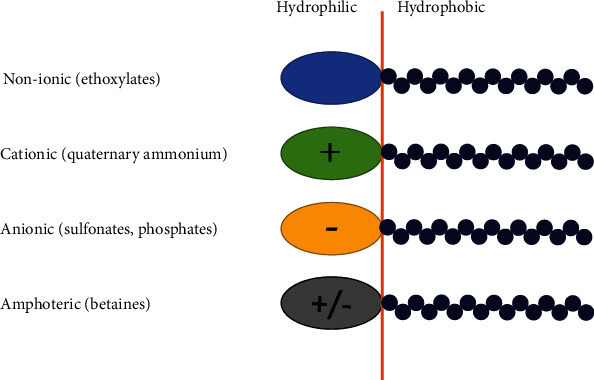
Surfactants types determined by the polarity of their head group.

The main criterion used to categorize chemical surfactants is the charge that remains on the hydrophilic heads of the molecules following dissociation in water, i.e., anionic, cationic, nonionic, and amphoteric as shown in Figure 3. The amphiphilic nature of chemical surfactants, in addition to their other distinctive qualities, makes it possible for these substances to be widely used in various modern cosmetic and personalized skincare products [31, 32].

Sodium-lauryl-sulfate (SLS), sodium-dodecyl-sulfate (SDS), sodium lauryl ether sulfate (SLES), cocamide-di-ethanol-amide, and cocamidopropyl-betaine are commercially available chemical surfactants. These chemicals are most commonly used in cosmetics and personal cleaning products [33]. Several chemical surfactants are capable of executing several actions, including [29, 34, 35],

- removing dirt from the body surface
- enhancing lather foaming in shampoos
- emulsification of non-compatible liquids
- conditioning the skin and hair, and
- moisturizing and wetting the skin and hair

In addition, they can absorb liquids and lower the surface tension of water. Even though these products provide a wide variety of beneficial effects, it has been anticipated that regular application of chemical surfactant-based personalized care and cosmetic products may have detrimental consequences for people [36, 37]. The most noticeable effects of these chemicals include abnormalities in the skin's microbiota, inflammation, and other allergic reactions, which may be brought on by the reaction between chemical surfactants and the skin's epidermal layer [6, 38]. It is not quite clear what exact mechanisms are responsible for the potentially harmful consequences of chemical surfactants. On the other hand, it is thought that these adverse effects are caused due to the chemical surfactant properties (physical and chemical), the applied concentration, and the total time of contact for the epidermis to interact with the substance [39].

Keratinocytes are the primary cells that make up the skin's epidermal layer, which is also called the dermis. When keratinocytes undergo the final stage of differentiation, called terminal differentiation, they form corneocytes. Corneocytes make up the outermost layer of the epidermis, which is called the stratum corneum [40]. These cells are protected and made rigid by being surrounded by a matrix rich in lipids and a cell envelope made of tough, cross-linked proteins [41]. Desmosomes keep corneocytes closely connected to each other. The intracellular spaces of the cells that make up corneocytes contain various types of lipids [42]. In a sample, the lipid ratios are 10% free fatty acids, 10% cholesterol esters, 25% free cholesterol, and 50% ceramides. These lipids help regulate transepidermal water loss and are known as natural-moisturizing factors [43, 44]. Chemical surfactants can disrupt the balance of these intracellular lipids and cause the denaturation of proteins in skin cell membranes, a process known as delipidation [45]. This can also lead to acute swelling of the stratum corneum, followed by deswelling. Chemical surfactants can also negatively affect the immune cells living in the skin, such as Langerhans cells and keratinocytes, and can impact immune responses [46–48].

The likelihood of chemical surfactants penetrating through skin layers and causing denaturation of proteins, hypersensitivity, or skin inflammation is greatly influenced by the physical nature of the surfactants in solution (whether they exist as monomers or micelles) as well as their concentration [46, 49]. Micelles are produced once monomers of surfactant aggregate at a specific concentration and temperature in a solution. This surfactant concentration is called the critical micelle concentration (CMC) [50]. Chemical surfactants, monomers, and micelles permeate skin layers and cells; however, due to their unstable nature, micelles disintegrate after contact with the skin. Some studies have demonstrated that when the critical micelle concentration (CMC) is attained, the significantly larger micelle size and surface actions reduce surfactant uptake across skin layers [51, 52]. However, other studies reported that chemical surfactant monomers and micelles invade associated cells and skin layers [53, 54]. However, during the micelle synthesis and breakdown process, smaller micelles (submicelles) are formed, which may be able to permeate the skin.

Furthermore, current advancements in biological sciences have enhanced research potential to explore the natural sources for surfactant production, improving skin health, and solving the earlier-mentioned issues. In light of the above, the application of biosurfactants has emerged as a potentially useful option [55].

## 3. Plant-Based Biosurfactant

Due to its reliance on carbon sources, the higher production cost and potential impact on food security make manufacturing biosurfactants less prevalent. Plants are widely considered a significant origin of natural surfactants due to their ability to produce unlimited bioactive and biodegradable chemicals. These naturally occurring chemicals are less toxic and harmful than synthetic/artificial chemicals. Saponins are a class of bioactive chemical compounds found in plants [12]; they are so named because they produce a soapy lather when combined with water and agitation. They must come from natural sources to ensure they are nontoxic, biodegradable, and environmentally friendly. Previous research [56] indicates that the physicochemical properties of natural saponins surpass those of synthetic saponins. Plants rich in saponin possess desirable biological and physicochemical attributes, making them a potential natural biosurfactant source for academic and industrial applications. Nowadays, saponins are still principally derived from a variety of plants, such as *Saponaria officinalis*, oleandrin, foxglove, soapbark tree *Quillaja saponaria*, licorice, and horse chestnuts [57].

### 3.1. Saponins

Saponins are amphiphilic glycosides that occur naturally and are composed of polar glycone moieties or sugars that are distinct in structure from nonpolar aglycone-structure moieties, also known as sapogenins [58]. The aglycone counterparts of saponins categorize them into steroidal and triterpenoid saponins. The primary difference between the two classes is the number of carbon atoms, with steroidal saponins having 27 and triterpenoid saponins having 30 [59]. Triterpenoid saponins are further classified into oleanane, ursolic acid, and dammarane saponins, while steroid saponins are classified as furostanol and spirostanol types. Steroidal glycoalkaloids comprise the aglycone backbone of saponins from the Solanaceae family [60].

Based on the number of sugar units, saponins are classified into three categories: monodesmosidic, bidesmosidic, and tridesmosidic saponins. Monodesmosidic saponins have one sugar unit attached to carbon-3; bidesmosidic saponins have two sugar units attached to C-3 and C-26 or C-28, while tridesmosidic saponins consist of three sugar units attached. The sugar chains can be branched or linear and typically contain glucuronic acid (GlcA), D-fructose (Fuc), D-xylose (Xyl), L-rhamnose (Rha), L-arabinose (Ara), D-galactose (Gal), and D-glucose (Glc) [61, 62]. Saponins are a diverse class of secondary metabolites found in over a hundred vascular plant families and some marine sources. Triterpenoid saponins are mainly derived from dicotyledonous plants such as the *Fabaceae*, *Araliaceae*, and *Caryophyllaceae* families, while steroidal saponins are primarily found in monocotyledonous plants such as the *Liliaceae*, *Dioscoreaceae*, and *Agavaceae* families. Saponins are present in various plant parts, including the stems, roots, leaves, fruits, pericarp, flowers, and seeds, with their composition and concentration varying significantly between different plants and even within the same plant in different parts. The growth environment of the plant and the extraction process also affect the amount and composition of saponins extracted from a plant [63].

### 3.2. Saponin Surfactants and Applications

The amphiphilic structure of saponins, comprised of a nonpolar aglycone (lipophilic) and a polar glycone moiety (hydrophilic), gives them surfactant properties in aqueous solutions. These properties are because saponins are dissolved in water. This structure is similar to synthetic surfactant molecules with a hydrophilic head group that dissolves in water and a hydrophobic tail group that does not. Surfactants are categorized based on the charge of their hydrophilic polar head group, which can be nonionic, zwitterionic, cationic, or anionic [64]. Saponins belong to the nonionic surfactant category because their hydrophilic portion comprises sugar chains (water-soluble), while their hydrophobic portion can be steroid or triterpenoid (water-insoluble). Saponins, which are nonionic surfactants, have a wide range of surfactant properties, including emulsification, wetting, detergency, foaming, micellization, and surface activity [65]. These properties make saponins potential natural alternatives to synthetic surfactants in personal care products, detergents, and agricultural formulations. Moreover, saponins have been studied for their antimicrobial, anticancer, and anti-inflammatory properties, making them a promising area of research for developing new cosmetic formulations, drugs, and therapeutics [66].

## 4. Microbial Biosurfactant

It has been reported that various microorganisms, such as bacteria, fungi, and yeasts, are capable of producing biosurfactants in an effective manner. However, the type of microorganism used, the composition of the media, the characteristics of the substrate, and other intrinsic and extrinsic factors during microbial culture growth all play an essential role in determining the quantity and quality of biosurfactants produced [67, 68]. The first step in the process of producing biosurfactants is to choose an appropriate strain of microorganism. When nutrient conditions are inadequate, biosurfactant synthesis can take place either inside the cells of the microbe or outside of them [69]. It can take place during the exponential phase of growth or the stationary phase of growth. The nature of the biosurfactant is also determined by the source of the microorganism and the isolation techniques employed. For example, an isolated strain obtained from a polluted site is more suitable for breaking down a particular contaminant, as it can utilize as an energy source or substrate. Conversely, other microorganisms are incapable of surviving or generating surfactants [70]. In addition to boosting the carbon uptake from the soil, biosurfactants play a physiological role in enhancing the bioavailability of hydrophobic molecules engaged in cellular signaling or maturation processes. Biosurfactant production in polluted environments is thought to increase cellular motility, decrease surface tension at the phase barrier, and improve nutrient absorption from hydrophobic substances; however, the fundamental physiological mechanisms are still being explored [70]. Developing quick and effective approaches for screening microbial spp., evaluating their emulsification properties, and their ability to reduce interfacial or surface tension are crucial factors in exploring biosurfactant molecules. Bushnell and Haas [71] first documented biosurfactants produced by *Corynebacterium simplex* and *Pseudomonas* sp. cultured on minimum media with paraffin, mineral oil, or kerosene as carbon sources. Another example of BS discovery is apparent in the work done by Jarvis and Johnson in 1949 when they deciphered the structure of the rhamnolipids found in *Pseudomonas aeruginosa* [72]. Subsequently, in 1968, Arima and coworkers described a novel molecule produced by the bacteria *Bacillus subtilis* with strong surface activity; they called it surfactin [73]. Since then, a significant number of microbial strains, like bacteria, fungus, and yeast, have been documented that are capable of producing biosurfactants in large quantities.

## 5. Low- and High-Molecular-Weight Biosurfactant Molecules

There are two primary categories of biosurfactants: those with a high molecular weight and those with a low molecular weight. The most prominent examples of these include rhamnolipids, sophorolipids, and threhalolipids, although mannosylerythritol lipids and surfactin are also significant bioemulsifiers. The hydrophobic tail is composed of one or more fatty acid chains, which may be branched, while the hydrophilic head group is composed of a phosphate group (in the case of phospholipids), a peptide loop (in the case of lipopeptides), or a sugar moiety (in the case of glycolipids). For example, glycolipids can be monosaccharide rhamnose or disaccharides. Although biosurfactants with high molecular weight are superior as emulsifying agents, low-molecular-weight BS are superior in lowering interfacial and surface tensions [29]. Each category is further subdivided into additional classifications based on the chemical composition of its constituent components. Phospholipids, glycolipids, lipopeptides, and lipoproteins are the primary categories of low molecular weight biosurfactants [24, 74]. Conversely, biosurfactants with high molecular weight comprise sugar surfactants (abbreviated as CiGj), which can be derived by combining biosourced sugar head groups and fatty acids through esterification. They can also be obtained from microbial polymeric biosurfactants, as discussed in studies conducted by Avila-Arias et al. [75] and Hellweg et al. [57]. However, the main focus of this review will be on low-molecular-weight BS, specifically glycolipids and lipopeptides.

Glycolipids are prevalent among biosurfactants and are considered one of the most promising types for applications in cosmetics, pharmaceuticals, and food sectors [38]. The MELs (MELs A-C) and sophorolipids (lactonic and open acidic) of *Candida* spp., the trehalose lipids of *Rhodococcus* and *Mycobacterium* spp., rhamnolipids (mono- and di-rhamnolipids) of *Pseudomonas* spp. are all examples of glycolipids [15, 19], as mentioned in Table 1. Since sophorolipids have great stability throughout a wide range of pH, temperatures, and salinities, they are increasingly being considered a competitive alternative to surfactants derived from petroleum. They are effective against microbes, and they also hydrate nicely, froth effectively, and work as emulsifiers [76]. The production of lipopeptides is predominantly attributed to *Bacillus* species [77]. The hydrophilic end of lipopeptides comprises seven to ten amino acids, while its hydrophobic end is typically made up of fatty acids that are organized in either linear or cyclic sequences [78].

## 6. Exploring the Interplay between Biosurfactants, Skin Microbiome, and Human Skin Health

It is believed that one square centimeter of human skin contains approximately one billion different types of microorganisms [52, 82]. These microorganisms include bacteria, fungi, viruses, and yeast. Previous studies [83] have indicated that newborn infants are devoid of microorganisms prior to birth. However, the introduction of microorganisms takes place during the process of labor and delivery as well as after birth. The process of birth, whether normal delivery or surgical delivery, affects the microbial diversity of a new-born's epidermis (caesarean section). Babies born via vaginal delivery have a microbiota analogous to the microbial community that lives in their mother's birth canal [84]. In contrast, babies born via caesarean section have a microbiota analogous to the microbial population living on their mother's skin surfaces [85]. After initial colonization, the skin's microbial diversity is typically further expanded by continuous exposure to atmospheric microorganisms, regulated by T lymphocytes, and related host traits like sexuality, environment, nutrition, location, and the usage of personal and cosmetic products [86, 87].

It has become abundantly evident that bacteria are the microorganisms that are most commonly found on the skin, and the inter- and intrapersonal linked bacterial varieties are practically identical [88]. In a study, 19 bacterial phyla were found in 20 different skin areas of 10 healthy people using 16S rRNA genotyping. The majority of the sequences were classified into the following four phyla as their characteristics: Bacteroidetes (6%), Proteobacteria (17%), Firmicutes (24%), and Actinobacteria (52%) [89, 90]. They reported the prevalence of *Cutibacterium* and *Staphylococci* species in small oil-producing glands present in the skin of mammals (sebaceous areas), while *Corynebacteria* spp. They have predominated in moist areas. Several different kinds of bacteria have been detected in the skin's dry areas, including the hypothenar, volar-forearm, and buttocks [91].

Furthermore, culture-independent (16 s rRNA) and culture-dependent approaches were used to identify the four major phyla mentioned earlier from the two young individuals' volar-forearms [92]. Even though a very small percentage (around 9.7%) of Gram-ve cells were also exhibited in samples, in addition, 16 s rRNA sequencing was the primary method used to identify those Gram-ve bacterial cells [82, 93]. The immune system of the skin is highly reactive and has the ability to control both harmful and beneficial bacteria. The skin serves as its own self-contained ecosystem. However, the notion of enhancing a healthy skin microbiome through the addition of bioactive chemicals in beauty products, such as microbial biosurfactants, has been suggested for quite some time [37, 38].

Biosurfactants possess important physiochemical qualities that are beneficial for maintaining healthy skin. For example, the fatty acid endings of their molecules are useful for hydrating the rough and dry surfaces of the skin. In addition, the available fatty acids have the potential to act as antioxidants, which would stop the production of free radicals caused by UV light [24, 94]. In addition, the breakdown of triglycerides available in microbial biosurfactants into fatty acid chains by *Cutibacterium acnes* may enable to maintain the skin's pH at an acidic level, which in turn encourages the adhesion of indigenous microbial flora on the skin and prohibits the development of pathogenic bacteria, thereby helping to sustain a healthier skin microbiota [4].

In contrast to chemical surfactants, biosurfactant components, which include biomolecules (i.e., polysaccharides, triglycerides, and proteins), are quite analogous to cell membrane components of the skin [1]. Furthermore, the mobility of molecules through the skin cell membrane is determined by lipophilicity and surface interaction [80]. As a result, the distinct composition of biosurfactants allows them to mobile with high rate of permeability across the skin cell membrane, which allows them to regulate skin barrier properties and activate favorable effects associated with hair regeneration and skin repair processes. In addition, rhamnolipids, sophorolipids, MELs, and surfactants have all been shown compatibility with human skin in in vitro experiments [95, 96]. Likewise, the chemical nature of biosurfactants makes them desirable due to their foaming, emulsifying, solubilization, and wetting properties, which make them suitable for usage as constituents in powders, creams, shampoos, lotions, and other key cosmetic items [1]. The performance of these products is influenced by the chemical makeup of the components they contain. Some examples include RelipidiumTM, a moisturizer for both the body and face made in Monheim, Germany, by BASF; SopholianceTM S, a line of deodorants, face cleaners, and shower gels produced in Paris, France, by Givaudan Active Beauty; and Kanebo skincare products, which include moisturizers, cleansers, and UV filters made by Kanebo Cosmetics in Tokyo, Japan, are examples of skincare and commercially available cosmetic products that contain biosurfactants from microbial origin [97]. Ganesan et al. [98] utilized a partially purified surfactant created by *Bacillus subtilis* to develop a stable nanoemulsion in a recent investigation. They combined xanthan gum with the nanoemulsion to create a thickened cosmetic emulsion that displayed pseudoplastic behavior without compromising its stability. This thickened nanoemulsion has potential applications in a variety of cosmetic products.

Recently, Etemadzadeh et al. [99] reported that the biosurfactant produced by salt-tolerant *Bacillus halotolerans* exhibits multiple clinically significant characteristics and can serve as a raw material in food, medical, and cosmetic product formulations. In vitro studies on the isolated lipopeptide BS showed that it had antibacterial and antioxidant characteristics with an effectiveness rate of 90.38 percent when present at a concentration of 0.8 mg/mL. Additionally, it displayed anticancer activity by inducing apoptosis in MCF-7 cells while having no harmful effects on normal HEK-293 cells. In another study, Abed Almjalawi and Al Sa'ady [100] reported the impact of *Bifidobacterium* species-produced BS cytotoxicity on the WRL68 normal cell line and the MCF-7 cell lines. Their findings revealed that the produced BS displayed varying degrees of cytotoxic activity towards MCF-7 and had the least impact on WRL68 cell lines. The researchers recommended the utilization of these produced BS in healthcare products. Adu et al. [6] compared the effects of naturally derived glycolipid biosurfactants (sophorolipids and rhamnolipids) with synthetic surfactants (sodium lauryl ether sulfate) on human keratinocyte cells, which are important for skincare applications. According to the findings, cell viability and production of inflammatory cytokines are only slightly affected by acidic sophorolipids and mono-rhamnolipids, while the effect of different glycolipids on cells varies relying on their chemical composition. Additionally, at noninhibitory concentrations, di-rhamnolipids were found to significantly reduce inflammation and increase the expression of anti-inflammatory cytokines, making them a potential substitute for synthetic surfactants in skincare formulations and useful for topical skin infections like psoriasis.

Alpha- and beta-defensins, enzymes, and bacteriocins, are examples of naturally occurring inhibitory chemicals that can be found on the skin surface [101]. These chemicals protect the skin microbiota and skin from infections. In addition, microbial biosurfactants have been claimed to have the potential to be beneficial in the treatment of skin problems. *Staphylococcus aureus*, *Pseudomonas aeruginosa*, *Candida acnes*, and *Streptococcus pyogenes* are some skin pathogens inhibited by several different biosurfactants, which have been shown to have efficient inhibitory mechanisms. Due to this, biosurfactants have been proposed as a potential alternative to traditional antibiotics, even though the bactericidal action of biosurfactants is often weak and varies greatly from one molecule to another [102].

## 7. Antimicrobial Properties of Microbial Biosurfactants

The pharmaceutical industry introduced over 20 different types of antibiotics worldwide between 1930 and 1962, resulting in a significant decrease in mortality rates associated with bacterial-related infections [97]. However, in the last 50 years, the discovery and large-scale production of antibiotics have significantly declined, with only three new antibiotic classes, namely, lipopeptide daptomycin, oxazolidinone linezolid, and mupirocin, added in recent times [103, 104]. Biosurfactants have been found to possess antimicrobial properties that affect the permeability of cell membranes, with microbial extracellular glycolipids being used as a reagent to treat cancer cells due to their anticancer activity. *Candida bombicola's* BS (sophorolipid surfactants) have virucidal and spermicidal activity with antiadhesive properties that prevent pathogenic microbe adhesion to infected areas or solid surfaces. *P. flocculosa's* flocculosin has antifungal properties against human mycose and pathogenic yeast [105]. Antibiotics have a critical role in the treatment and control of many epidermal bacterial infections; however, the overuse of antibiotics has led to the development of antibiotic resistance [97], necessitating the development of more effective antimicrobial drugs [106, 107]. Numerous investigations have shown that biosurfactants have antibacterial effects, such as sophorolipids produced by *Starmerella bombicola*, rhamnolipids produced by *P. aeruginosa*, surfactin by *B. subtilis*, glycolipids by *Ustilago* and *Aspergillus*, and other lipids extracted from *Rhodotorula*, as presented in Table 2 [97]. Kumari et al. [108] have explored techniques for the low-cost production and design of biosurfactants from *Brevibacterium casei* strain LS14. The purified biosurfactant was identified as a lipopeptide biosurfactant with efficient antibacterial activity against *Pseudomonas aeruginosa* due to free radical scavenging activity and oxidative stress. Cellular cytotoxicity was also observed in a dose-dependent induction of apoptosis. Kubendiran et al. [109] executed a study in which they formulated an ointment for cutaneous infections by combining *Tridax procumbens*-infused oil and gelatin-stabilized Ag NPs with a biosurfactant obtained from *Lactobacillus casei* (MT012285). The ointment displayed increased ability to reduce microbes present in clinical pathogens such as *K. pneumoniae, P. aeruginosa, S. aureus, and E. coli*. It caused minimal damage to human red blood cells. Additionally, when used in higher concentrations (4.0 mg/mL), the ointment demonstrated reduced levels of cytotoxicity on the fibroblastic cell line (L929) and promoted high cell migration rate, indicating its potential use as a topical agent for wound treatment and as an antimicrobial agent.

The stability of the microbial communities at the various areas throughout the human body and the maintenance of the proper balance of these communities are directly associated with the surface active agents produced by these microorganisms. It was recognized that the microbial communities in many parts of the body are interconnected, including the mouth and the genital tract, and even the gastrointestinal tract and the genitourinary system [110]. As a result, scientists [110, 111] demonstrated that bacteria are able to travel through the digestive tract and exchange ecological habitats with the intestines. Additionally, they found correlations between the vagina and the oral cavity. The mouth cavity is home to the microbiota most prone to change, whereas the bacteria found in the stool and vagina are the most consistent [112].

Consequently, the discharge of bioactive compounds, such as biosurfactants, might be an essential component in preserving a niche. For instance, the stability of a Lactobacilli community in the vaginal environment is associated with the overall health of the area [113]. Although, *Lactobacillus* strains capable of making biosurfactants are not commonly found in the vaginal environment [114]. However, biosurfactant molecules have the potential to disperse throughout the environment and alter the surface tension (ST), hence preventing the growth of infections.

According to Brzozowski et al. [115], *Lactobacillus* spp. can produce biosurfactants mostly made up of polysaccharides, phosphate, and amino acids in varying proportions. These biosurfactants are primarily categorized as glycolipids or glycolipoproteins. The molecules also have an antifungal and antibacterial effect against *Candida albicans* and pathogenic bacteria, respectively, which have the potential to cause disease, including *N. gonorrhoeae*, *K. pneumoniae*, *E. aerogenes*, *S. saprophyticus*, and *E. coli*. However, *Lactobacilli* sp. can also be found in other body parts, i.e., the skin, intestines, mouth cavity, and gastrointestinal tract (GI) [116]. The GI tract normally has a stable microbiota community, but numerous bacteria that produce biosurfactants and are present in consumable food can reside in the GI tract, i.e., *L. paracasei* subspecies *paracasei*-A20 from Portuguese dairy products [117]. The biosurfactants produced from this strain possess high antibacterial and antiadhesive effects against various bacteria and fungi.

Moreover, Iram et al. [118] and Liu et al. [119] reported that *L. fermentum*, *L. pentosus*, and *L. acidophilus*, which were isolated from human milk, fruits, milk products, and fermented shrimps in Malaysia, have the ability to produce cell-free biosurfactants that exhibit antibacterial properties. Haakensen et al. [120] identified *Pediococcus dextrinicus* SHU1593 as another bacterium capable of producing biosurfactants. The lipoprotein biosurfactants produced by this strain are cell bound and possess antibacterial effects against bacteria that cause human food poisoning, such as *S. typhimurium*, *E. aerogenes*, and *B. cereus*. Merghni et al. [121] suggested the use of cell-associated biosurfactants produced by *L. casei* ATCC393 and *L. casei* LBI for treating oral diseases caused by *Staphylococcus aureus*, due to their antimicrobial and antibiofilm properties. Other bacteria in different parts of the human body, as well as those belonging to the *Lactobacillus* genus, have been found to produce antimicrobial biosurfactants, according to Ferreira et al. [1], Fraszczak et al. [122], and Merghni et al. [121]. These biosurfactants have the potential to inhibit microbe growth. *Pseudomonas aeruginosa* is among the most prominent producers of biosurfactants among these microorganisms; for example, *P. aeruginosa* ATCC10145 produces cell-free rhamnolipid biosurfactants that have antibacterial and antifungal properties [123].

Scholars [24, 96] have identified the most significant group of antimicrobial biosurfactants derived from bacteria that are associated with human health, including glycolipoproteins, glycopeptides, glycolipids, and lipopeptides. Approximately 16 research papers have been published on the production of *Lactobacilli* antimicrobial biosurfactants [124], with ten characterized as cell-free biosurfactants and seven characterized as cell-associated biosurfactants. De Giani et al. [124] reported that small glycolipids and lipopeptides were produced by five of the cell-free biosurfactants, such as *L. acidophilus* NCIM2903 and a *Lactobacillus* strain from curd. In contrast, cell-associated antimicrobial biosurfactants are more complex and can be identified by their multiple constituents, such as glycolipoproteins. However, information on conformations, molecular weight, and modes of action is still lacking [102].

Biosurfactants also possess antibiofilm properties, which is an additional beneficial antibacterial characteristic [28]. Biofilms are an adaptive mechanism and survival tactic commonly used by bacteria. The extracellular polymeric substance (EPS) in the biofilm serves as a shield for the bacteria, protecting them from harmful environmental factors and immunological responses. Chemical gradients in the biofilm allow bacteria to survive in various physiological conditions, providing protection in different environments. Skin diseases such as chronic wounds, impetigo, and acne vulgaris are associated with biofilms [125]. Several studies conducted in vitro and in vivo [47, 125] have linked biofilms to various skin infections. These studies have revealed the biofilm-producing potential of *Staphylococcus aureus*, *Cutibacterium acnes*, *Escherichia coli*, and *Pseudomonas aeruginosa*, which cause skin infections. These bacterial cells are embedded in a matrix of extracellular polymeric substances (EPS), which consist of extracellular DNA, lipids, polysaccharides, and proteins.

Researchers reported microbial biosurfactants' effectiveness during several in vitro investigations, including suppressing new biofilms formation, preventing surface adhesion, and destroying biofilms that have already been formed [28, 50, 125].  Table 3 summarizes the multiple studies conducted to investigate the broad spectrum of microbial surfactants and their possible uses in the cosmetic sector, having various antimicrobial and other activities.

Karlapudi et al. [145] reported that the glycolipid biosurfactant produced by the *Acinetobacter* M6 strain possesses an inhibitory effect at 500 g/mL concentration. They found that the glycolipid biosurfactants could reduce biofilm formation by 82.5% in MRSA bacteria. In a study with *E. coli* CFT073, Rivardo et al. [146] found that *B. subtilis* V9T14 produced a biosurfactant having a biofilm inhibitory effect and reduced formed biofilm by 97%. In addition, the rhamnolipid biosurfactants that *P. aeruginosa* produces during biofilm synthesis are essential for maintaining channels that allow fluids to move through biofilms [20]. These channels are maintained by affecting cell-cell interaction and the attachment of bacterial cells to surfaces. In addition, rhamnolipids can induce biofilm detachments and dispersals, which ultimately make cells more open to attack by antimicrobial agents. The probable presence of toxins in *P. aeruginosa* strains that produce rhamnolipids makes it difficult for these rhamnolipids to be produced on a large scale or accepted for use in food, cosmetics, or pharmaceuticals. This is because of the pathogenic nature of these strains. Despite this, there is a growing interest in developing biosurfactants from nonpathogenic microorganisms, i.e., probiotics and prebiotics bacterial producers [74, 124].

## 8. Biosurfactants as a Moisturizer for the Skin's Surface

Although shampoos and other personalized care products can provide short-term benefits, prolonged contact with the body surface can harm the outermost layer of the skin (stratum corneum), making cellular lipids more soluble and denaturing proteins [9, 40]. To address this issue, microbial origin biosurfactants have been developed as an alternative to chemical surfactants, with the added benefit of effective skin moisturization and compatibility as a skin applicant [20, 22, 31]. In a recent study, Mawani et al. [147] formulated an antidandruff shampoo using a combination of biosurfactants called mannosylerythritol lipids (MEL) and sodium lauryl sulfate (SLS), along with antidandruff agents salicylic acid and benzoic acid. The shampoo's physicochemical parameters were evaluated, and it showed antimicrobial activity against *Staphylococcus aureus* and *Malassezia furfur*. The combination of MEL and SLS, along with other antimicrobial agents, improved the antidandruff properties of the shampoo, indicating that SLS can be replaced by a combination of biosurfactants to reduce the use of chemical surfactants.

**Figure 4:**
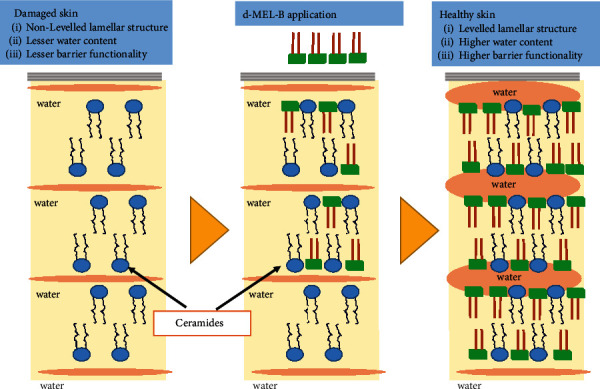
Effects of biosurfactants (MEL) application on rough skin and MEL effect to make skin healthy.

Specifically, MELs produced by different *Candida* species have been shown to improve the cell viability of pretreated skin models, with MEL-A (a glycolipid) improving the viability of a damaged and dried 3D skin model by approximately 90% after 24 hours of incubation [38]. In addition to biosurfactants, ceramides—a type of epidermal lipid—play an important role in establishing the skin barrier and retaining moisture in the epidermis. Studies conducted by Kiran et al. [28] and Tucker et al. [12] have revealed that skin disorders such as dry, itchy, and inflamed skin and scaly patches, typically found on the knees, elbows, trunk, and scalp, are caused by a lack of ceramides in the stratum corneum, which leads to thickening of the skin's top layer. While ceramides, whether natural or synthetic, can effectively reduce skin roughness, their production is quite expensive. Therefore, MELs that possess comparable qualities can be a viable and cost-effective alternative. In addition, MELs have been shown to hydrate the skin, retain water, smooth rough skin, and restore damaged skin cells, as depicted in Figure 4 [28].

Including MELs in cosmetic products is primarily done to enhance the ability of the stratum corneum to retain moisture and repair hair damage (as shown in Figure 4). Aquaporins (AQPs), a group of proteins responsible for creating water channels in the cell membranes of humans, plants, and microbes (as reported by [148]), play a crucial role in regulating various skin factors by allowing the movement of water and other small solutes such as glycerol and urea through the epidermis. Mammals possess thirteen AQPs (0-12), with AQP-3 being the most studied due to its high presence in human skin. AQP-3 allows not only water but also other noncharged solutes like glycerol and urea to be transported. This allows it to maintain the epidermis's water balance while facilitating the transfer of small solutes.

Bae et al. [149] found that 95% purified glycolipid (MEL-B) can restore epidermal barrier function and reduce the downregulation of UV-induced AQP-3 in keratinocytes. This suggests that MELs could be used in skincare products to keep the skin microbiota healthy. The antioxidant and protective properties of MEL-C against H_2_O_2_-induced oxidative stress in human skin fibroblasts were described by Takahashi et al. [150]. The results showed that at a dose of 10 mg/mL, MEL-C had the maximum radical sequestration efficiency of all glycolipids (50.3%). The overproduction of melanin is what leads to hyperpigmentation, which includes things like freckles. MELs usage in skin-whitening formulations, on the other hand, has shown promise in inhibiting the formation of melanocytes and enhancing the skin's complexion.

Moreover, sophorolipids have the potential to promote hair restoration and protect the skin, and Kao Co. Ltd. in Japan is a leading manufacturer of these compounds for use as a humectant in various industrial products such as skin moisturizers, hair moisturizers, and lipsticks [135]. Additionally, there is a hypothesis that sophorolipids can decrease subcutaneous fat in the skin by increasing leptin production in adipocytes. Similarly, rhamnolipids are considered biocompatible and are a promising ingredient for use in pharmaceutical formulations of cosmetics and personal skincare products, as suggested by several studies [19, 20, 27, 151]. It is also speculated that moisturizers containing lipid biosurfactants may enhance the product's ability to penetrate the skin deeply, leading to the production of new collagen and better management of factors that contribute to skin structure deterioration.

## 9. Studies on the Effects of Cytotoxicity

The utilization of chemical substances in cosmetic production can lead to a challenge, which is the inclination of these components to incite allergic reactions. According to an epidemiological study [152] conducted in the United Kingdom, nearly 23% of females and 14% of males develop an adverse response to the cosmetic and personalized care products that people utilize during a particular period. Additionally, around 10% of these negative effects were allergic reactions. Aluminium-based compounds can be found in various personalized care products, including antiperspirants and deodorants, which are among the most widely used [2]. In some cases, it has been suggested that one of these compounds contributes to Alzheimer's disease [153]. However, there is insufficient scientific evidence to support these hypotheses. In some studies, it has also been observed that one of these compounds is a contributing factor in Alzheimer's disease. However, Bouslimani et al. [154] reported a significantly negative impact of polypropylene glycol- (petroleum-based compounds) based skin care products (antiperspirant) on the metabolism of skin microbiota. These polypropylene glycol-rich molecules have a 1.9-week half-life on the skin. Utilizing biosurfactants may be able to rectify these possible flaws, even though they may be quite minor.

In order to be considered for use in the cosmetic and health care industries, microbial biosurfactants must demonstrate low levels of toxicity, which means they should not cause irreversible damage to skin cells. Shao et al. [155] tested ten sophorolipids with varied molecular compositions on human esophageal cancer cells. The research showed that diacetylated sophorolipids were more hazardous than monoacetylated ones (MIC = 30 g/mL vs. 60 g/mL) [155]. Another analysis showed that sophorolipid blends having 40.12% diacetylated sophorolipids were cytotoxic to epidermal fibroblasts at doses > 50 *μ*g/mL [156]. Nevertheless, sophorolipids at lower concentrations (0.5-5 g/mL) benefited wound healing and suppressed proinflammatory cytokine production in LPS-stimulated macrophages. The cytotoxicity of rhamnolipids isolated from *Pseudomonas* strain MCTG214(3b1) and *Marinobacter* strain MCTG107b towards HaCaT-cells, and transformed liver epithelial cells (THLE3) was found to be minimal up to a treatment concentration of 0.25 mg/mL, as reported by Voulgaridou et al. [157]. On the other hand, with as little as 0.002 mg/mL of treatment, synthetic surfactants showed harmful effects. Stipcevic et al. [158] found that biosurfactant toxicity increased with concentration. However, studies have shown that surfactin, MELs, rhamnolipids, and sophorolipids are safer for usage since they cause less damage to mammalian cells than their chemical analogues [12, 20, 27, 74]. To assess skin irritation and toxicity, scientists have conducted different types of research using both in vitro and in vivo methods [1, 20]. In recent years, there has been a shift towards using in vitro models due to advancements in technology, such as the development of 3D skin models that closely resemble human skin and possess barrier capabilities (Simms et al., 2020). These models have been improved overtime, including by increasing their shelf life and incorporating skin cells. While in vivo animal and human skin models were previously used in laboratory experiments, the development of in vitro skin models has provided alternative options. In vitro models of pig skin have also been developed and possess similar percutaneous uptake characteristics and permeabilization capabilities as human skin [159]. This has helped address the ethical concerns associated with using in vivo models.

## 10. The Effects of Biosurfactants on the Different Types of Skin Cells

*P. aeruginosa* is rarely found in healthy people, although it is prominent in patients with burns and chronic wounds. Psoriasin, an antimicrobial protein (AMP), is produced by epidermal keratinocytes in response to *P. aeruginosa* expressing flagellin antigen (Simms et al., 2020). Researchers Meyer-Hoffert et al. [160] reported that *P. aeruginosa* rhamnolipids could trigger psoriasin production even when they are not physically interacting with skin microbes or sensitive cells. Thus, these rhamnolipids limit pathogen colonization on the skin without affecting the skin microbiota or inflammatory responses. Furthermore, rhamnolipids have been reported to stimulate the growth of epidermal keratinocytes. According to the findings of Stipcevic et al. [161], rhamnolipids (di-RL BAC-3) at a concentration of 50 g/mL stimulated the proliferation of neonatal keratinocytes in the presence of medium containing serum, while at the same time, under similar conditions, it inhibited the proliferation of fibroblastic cells. Inhibiting fibroblastic cell differentiation is important for preventing tissue regeneration delays, and keratinocyte proliferation contributes to the reepithelialization of wounds; thus this action is important for the formulation of external wound healing cream. In addition, the proliferation of keratinocytes would help the reepithelialization of wounds. When the viability of fibroblastic cell lines was determined using the thiazolyl blue tetrazolium bromide (MTT) assay, the sophorolipids synthesized by hydrolyzing horse oil did not have a significant toxic effect at concentrations of up to 50 micrograms per milliliter. Interestingly, sophorolipids exhibited a stimulatory impact on fibroblastic cells even at low doses (0.1 g/mL). The primary components of horse oil are chemicals that are quite similar to those found on human skin. These include mono- and polyunsaturated (linoleic and palmitoleic) fatty acids. Maeng et al. [156] used this approach for the production of high-quality sophorolipids, beneficial for skin health.

Sahnoun et al. [162] experimented with SPB1 lipopeptide biosurfactant to determine the possible toxicity by administering it to mice in vivo. An LD50 value was set at 475 mg/kg. Mice who received subcutaneous injections of 47.5 mg/kg or less on a daily basis for 28 days showed no signs of mortality or abnormalities in behavior and motility. This included no signs of abnormal changes in behavior or locomotion. In addition, no cutaneous reactions, such as irritations, were seen in the study participants. The SPB1 did not affect hematological or serum quantitative results. The genotoxicity of surfactin C, a lipopeptide produced by *B. subtilis* BC1212, was evaluated through in vitro and in vivo experiments on ICR mice. The tests were conducted by administering a daily dosage of 500 mg/kg, and the results revealed that the compound did not demonstrate any indications of maternal toxicity, fetotoxicity, or teratogenicity in the mouse trials [158].

Researchers Senthil Balan et al. [163] evaluated the effect of Cybersan (glycolipid biosurfactant) had on 3 T3 embryonic-fibroblastic cells. This biosurfactant was derived from the *Cyberlindnera saturn* strain SBPN27. During the study, 97% cell viability was observed after 24 hours when the biosurfactant concentration was 200 ng/mL. In addition, the cell viability percentage was 92% at 400 g/mL, 60% at 600 g/mL, 79% at 800 g/mL, and 70% at 1000 g/mL concentration. This research evaluated Cybersan's antibacterial efficiency. These concentrations inhibited human clinical pathogens by a factor of 100. In addition, compared to other well-known glycolipid biosurfactants, the Cybersan biosurfactant had a significantly lower level of toxicity. Compared to their synthetically generated counterparts, biosurfactants are generally in a better position to be regarded as nontoxic substances.

## 11. Obstacles and Possible Approaches regarding Biosurfactant Applications

In order to be an effective cosmetic and personalized care product, they must fulfill key needs like moisturizing, protecting, cleansing, and preventing pathogenic microorganism infection [114]. Biosurfactants can replace chemically synthesized surfactants in cosmetics and other personalized skincare products if they perform effectively and are priced competitively. Some of these biosurfactants have been marketed as commercial products. (e.g., sophorolipids and rhamnolipids). However, molecular sequencing, engineering of metabolic processes, and microbial-enzymes applications are some new alternatives that can be used to optimize biosurfactants' yield and structural variability [20, 164]. As a result, we need to conduct thorough research and assessments in these areas. In addition, biosurfactants synthesized by microbes are often created as a blend of mixtures instead of a single element. This is because of the fact that microorganisms cannot synthesize single compounds and different compounds have different bioactivities. The pure form of individual compounds needs to be analyzed for their efficacy and the optimal dosages at which they can be applied [20, 24, 38].

Researchers [20, 33] believe that the pathogenicity of a few Gram-negative biosurfactant-producing bacteria, like *P. aeruginosa* (a Group II pathogen), is a major barrier to exploiting rhamnolipids from these bacteria for commercialization. This is an opportunistic-pathogenic bacterium, meaning it only causes infections when the conditions are favorable. More research needs to be done on their virulence factors, and certain precautions must be taken before their usage and mass production in formulations for cosmetic and personalized products can be guaranteed [20, 33, 95, 123]. It is significant that Evonik Industries in Germany was able to design a pathway for the production of rhamnolipids by metabolically engineered nonpathogenic host organisms. This development made it possible for rhamnolipids to be utilized in personalized care products in a cost-effective and risk-free manner [165].

Several studies [117, 166, 167] recommend the beneficial application of prebiotic and probiotic bacteria in cosmetics. Currently, prebiotics are more commonly used, while probiotics are mostly used as a cutaneous treatment or an added ingredient in various beverages [5, 50, 138]. Biosurfactants, which are useful in microbial biotechnology, have been obtained from specific types of organisms that generate probiotics and prebiotics. These biosurfactants are efficient at performing their intended tasks. Since the organisms used as probiotics and prebiotics do not harm the natural human flora, they could potentially serve as suitable replacements for biosurfactants produced by pathogenic organisms [168, 169]. Therefore, in order to exploit this new avenue, one would need knowledge of the genetic composition of organisms that produce probiotic and prebiotic biosurfactants, as well as the appropriate substrate and cultivation conditions for those organisms. It would make people more likely to use microbial biosurfactants in food, cosmetics, and other products for personal care.

## 12. Conclusion

The human skin plays a crucial role in the body's immune system, acting as a responsive ecosystem that interacts with the environment to maintain overall health. Despite its primary function as a barrier, the skin is constantly exposed to various exogenous elements, including toxins and infections, which can compromise its functionality [13, 75, 162]. Cosmetics and personalized care products can help nourish and protect the skin, but there is a growing interest in the potential of microbial biosurfactants to enhance the effectiveness of these formulations. Natural plant derived saponins have shown great promise as a potential source for biosurfactants due to their desirable physicochemical and biological attributes. These glycosides are amphiphilic, nonionic surfactants that display a range of surfactant properties, making them suitable for use in various applications, including personal care products, detergents, and agricultural formulations. The presence of saponins in various plant parts opens up the possibility of using them as a sustainable and renewable resource for the production of biosurfactants. However, further research is needed to fully understand the potential of saponins as biosurfactants and their broader applications in different fields. With more research, saponins may become a crucial component in the development of new and innovative products in the future. Studies have shown that biosurfactants produced by microorganisms have various advantageous features that can improve the qualities of cosmetics and personalized care products over the use of plant-derived saponins. These include their ability to emulsify oils, enhance the solubility of active ingredients, and moisturize the skin [37, 38]. Biosurfactants have also been shown to have antimicrobial properties, which can help prevent infections and other skin-related problems. While the mechanisms by which biosurfactants interact with the skin are not yet fully understood, ongoing research in microbial biotechnology, pharmaceutical research, and cosmetic science is expected to provide more insights into this area. This could lead to the development of new formulations that are more effective and better tailored to individual needs. Overall, the potential of microbial biosurfactants to enhance the effectiveness of cosmetics and personalized care products is an exciting area of research that has important implications for human health. As scientists continue to explore the many ways in which biosurfactants can be used to improve skin health, we can expect to see more innovative and effective products in the marketplace. By harnessing the power of microbial biotechnology, we can create a more sustainable and healthier future for ourselves and the planet.

## Figures and Tables

**Table 1:** Potential microorganisms used for production of biosurfactants [15, 79–81].

Microorganism	Biosurfactant
*Pseudomonas* sp.	Rhamnolipid
*Pseudomonas aeruginosa* strain *DS10–129*	Rhamnolipids
*Pseudomonas aeruginosa LBI*	Rhamnolipids
*Pseudomonas aeruginosa* strain *BS2*	Rhamnolipids
*Pseudomonas aeruginosa EM1*	Rhamnolipid
*Pseudomonas putida strain B17*	Rhamnolipids
*Pseudomonas* sp. strain *DSM 2874*	Rhamnolipids
*Pseudomonas aeruginosa SP4*	Rhamnolipid
*Candida bombicola* strain *ATCC 22214*	Sophorolipids
*Candida lipolytica* strain *IA 1055*	Sophorolipids
*Trichosporon asahii*	Sophorolipids
*Serratia marcescens*	Lipopeptide
*Serratia marcescens*	Lipopeptide
*Bordetella hinizi-DAFI*	Trehalose-2,3,4,2′- tetraester
*Bacillus subtilis*	Iturin, surfactin
*Rhodococcus* sp.	Extracellular lipids and glycolipid
*Candida* sp. *SY-16*	Mannosylerythritol (glycolipid)
*Candida* sp. *strain SY16*	Mannosylerythritol lipid

**Table 2:** Antimicrobial and other beneficial properties of different biosurfactant produced by microorganisms [38].

Biosurfactants	Bacterial strains	Properties
Lipopeptides	*Bacillus licheniformis*	Resistance to heat and the ability to emulsify the oils that are used in cosmetics
Pontifactin	*Pontibacter korlensis*	Antimicrobial, antibiofilm, and surface-active activities
Rhamnolipids	*Pseudomonas aeruginosa*	Excellent emulsification activity against various oils, ability to clear hydrophobic impurities, and nontoxicity
Serrawettins	*Serratia marcescens* SS-1	Lipopeptide surfactants lower surface tension
Pumilacidin	*Bacillus subtilis*	Antiviral for herpes simplex virus 1 (HSV-1)
Orfamide A	*Pseudomonas protegens*	Insecticidal against *Myzus persicae*
Rhamnolipid	*Pseudomonas aeruginosa*	Antifungals for *F. sacchari*
Pseudofactin II	*Pseudomonas fluorescens*	Disinfectant, antiadhesive
Sophorolipid	*Rhodotorula bogoriensis*	Antimicrobial
Emulsifiers	*Candida utilis*	Emulsifiers
Eremophilane derivative	*Microsphaeropsis* sp.	Antimicrobial
Glycolipoprote	*Aspergillus ustus*	Antimicrobial
Glycolipids	*Ustilago maydis* FBD12	Antimicrobial

**Table 3:** An overview of multiple studies conducted to investigate the broad spectrum of microbial surfactants and their possible uses in the cosmetic sector.

Biosurfactant type or producing organism	Research objective or application	Key findings	References
N-dodecyl asparagine (AS), sodium N-dodecyl tryptophan (TS), and sodium N-dodecyl histidine (HS)	To detect antimicrobial antidermatophyte properties activity *AS, TS*, *and HS*	(i) Antimicrobial activity against *Shigella dysenteriae*, *Bacillus cereus*, *E. coli*, *K. pneumoniae*, *S. aureus*, *Trichophyton mantigrophytes*, *Trichophyton rubrum*, *Candida albicans*, *Trichosporon cataneum*, and *Cryptococcus neoformans*	Fawzy et al. [126]
Emulsion of mannosyl erythritol lipid (MEL) biosurfactant with *Thymus vulgaris*, *Lippia sidoides, and Cymbopogon citratus* essential oil emulsions	To detect prepared emulsion antimicrobial activity	(i) *Antimicrobial* activity against *Escherichia coli*, *Staphylococcus aureus*, *Bacillus subtilis*, *Pseudomonas aeruginosa*, *Penicillium* sp., *Aspergillus flavus*, *fusarium oxysporum*, and *Candida albicans*	Zanotto et al. [127]
CATASAN produced by *Psychrobacter* sp. TAE2020	Antibiofilm and antibacterial	(i) Antibiofilm and antibacterial against *Staphylococcus epidermidis* (ii) Good emulsification activity in a wide range of pH and temperature	D'Angelo et al. [128]
Glycolipid-biosurfactant of *Shewanella algae* strain B12	Antibiofilm and antibacterial	(i) Antibiofilm and antibacterial agains*t* planktonic and biofilm forms of MRSA and antibiotic resistant *Acinetobacter baumannii*	Amirinejad et al. [129]
Rha-C_10_-C_10_ and Rha-Rha-C_10_-C_10_ of *Pseudomonas aeruginosa* SG*Δ*rhlC	Antimicrobial agents	(i) Antimicrobial activity against *B. wiedmannii* H238, *A. alternate* G2	Zhao et al. [130]
MA01 rhamnolipid of *Pseudomonas aeruginosa* MA01	Antibiofilm and antibacterial	(i) Shown positive Antibiofilm and antibacterial *activit*y against methicillin-resistant *Staphylococcus aureus* (MRSA) ATCC6538 bacterial cells	Saadati et al. [131]
Sophorolipids of yeast *Starmerella riodocensis*	Antimicrobial	(i) Positive antimicrobial activity against *Candida albicans* hyphal and biofilm formation	Alfian et al. [132]
Antimicrobial peptides (AMPs), acidic sophorolipids (ASLs)		(i) Antibiofilm property against *S. aureus* (ii) damages the cell membrane of *Escherichia coli* (*E. coli*)	Seena et al. [133]
Sophorolipids in combination with *palmarosa* essential oil	Antiacne product	(i) Biosurfacta shown antibacterial and antiacne activity against *S*. *aureus*, *S. epidermidis* and *Cutibacterium acnes* in cosmetic formulations	Filipe et al. [134]
Sophorolipids and rhamnolipids	Anticancer effects of glycolipids on skin cells	(i) Detrimental effect on melanoma cell viability compared to healthy human keratinocytes (application in sunscreens)	Adu et al. [135]
Lipopeptide biosurfactant surfactin (ITC/SF-LNC)	Anticancer effects of lipopeptide for topical treatment of skin carcinogenesis	(i) Suppressive effect on cytokeratins (ii) tumor growth inhibition (iii) recovery of skin architecture	El-Sheridy et al. [136]
Mannosylerythritol lipids (MELs) (MEL-A, MEL-B, MEL-C and MEL-D) of *Pseudozyma aphidis*	Antimicrobial and skin moisturizer	(i) *S. aureus* ATCC 6538 biomass disruption, reduction of the biofilm metabolic activity and a bacteriostatic/bactericidal effect (ii) enhanced moisturizing property	Ceresa et al. [137]
Mannosylerythritol lipids (MELs) BGC of *Moesziomyces antarcticus*	Antimicrobial and skin moisturizer	(i) Antimicrobial and skin moisturizer due to *LipA* and *LipB* genes	Liu et al. [138]
MELs (MEL-A, d-MEL-B, and MEL-C) of *Pseudozyma Antarctica*, *P. aphidis*, *P. rugulosa* and *P. parantarctica*	Skin moisturizer, restoring damaged cells	(i) MEL-A exhibited excellent moisturizing performance (ii) restored viability of the damaged cells (iii) d-MEL-B and MEL-C also efficiently restored the viability of the cells	Kitamoto et al. [139]
Mannan-fatty acid of *Candida tropicalis*	Biostimulation	(i) Recognized as key antigenic determinants	Kuraoka et al. [140]
BS1 and BS2 of *bacilli* strains and *Lactobacillus pentosus*	Antimicrobial and cytotoxic activity	(i) Antimicrobiala ctivity against gram-negative bacteria, not cytotoxic for fibroblasts (NCTC clone 929) (ii) cell-bound biosurfactant from *Lactobacillus pentosus* boost the growth of the fibroblast up to 113%	Rodríguez-López et al. [141]
Lipopeptide(s) of *pseudomonas* sp. OXDC12 strain	Antifungal, antibacterial, cytotoxic and antiproliferative activity	(i) Antigungal against *fusarium oxysporum*, *Candida albicans* and *Mucor* sp. (ii) antibacterial activity against *Staphylococcus aureus* MTCC96, *Salmonella typhimurium* NCTC 74, *Klebsiella pneumoniae*, and *Escherichia coli* MTCC1687 (iii) low-level cytotoxic and antiproliferative activities towards a few transformed cell lines, (i.e., RD, Hep-2 C, Vero and MCF-7) cell lines.	Chauhan et al. [142]
BS of *Lactobacillus acidophillus*	Cytotoxic activity, antibacterial	(i) 23% cytotoxic effect on breast cancer (AMJ-13) cell line (ii) have antibacterial activity against *S. aureus* and *E. coli*	Abdullah and Ismail [143]
Mannosylerythritol lipids (MELs) of *Pseudozyma spp.*	Moisturizing effects	(i) Moisturizing effects on human skin, moisturizing effects on human hair	Kitamoto et al. [139]
BS of *Chenopodium quinoa* and *Pseudomonas aeruginosa* UCP 0992	Emulsifying agents	(i) BS from *Pseudomonas aeruginosa* good performance, stability, and emulsification	Bezerraa et al. [144]

## Data Availability

The data used to support the findings of this study are included in the article.
